# Molybdenum Nanoscrews: A Novel Non-coinage-Metal Substrate for Surface-Enhanced Raman Scattering

**DOI:** 10.1007/s40820-016-0104-6

**Published:** 2016-08-31

**Authors:** Di An, Yan Shen, Jinxiu Wen, Zebo Zheng, Jun Chen, Juncong She, Huanjun Chen, Shaozhi Deng, Ningsheng Xu

**Affiliations:** State Key Laboratory of Optoelectronic Materials and Technologies, Guangdong Province Key Laboratory of Display Material and Technology, School of Electronics and Information Technology, Sun Yat-sen University, Guangzhou, 510275 People’s Republic of China

**Keywords:** Surface-enhanced Raman scattering (SERS), Molybdenum nanoscrews, Quasi-one-dimensional nanostructures, Electric field enhancements

## Abstract

**Abstract:**

For the first time, Mo nanoscrew was cultivated as a novel non-coinage-metal substrate for surface-enhanced Raman scattering (SERS). It was found that the nanoscrew is composed of many small screw threads stacking along its length direction with small separations. Under external light excitation, strong electromagnetic coupling was initiated within the gaps, and many hot-spots formed on the surface of the nanoscrew, which was confirmed by high-resolution scanning near-field optical microscope measurements and numerical simulations using finite element method. These hot-spots are responsible for the observed SERS activity of the nanoscrews. Raman mapping characterizations further revealed the excellent reproducibility of the SERS activity. Our findings may pave the way for design of low-cost and stable SERS substrates.

**Graphical Abstract:**

Mo nanoscrews are for the first time cultivated as a novel type of SERS substrate. The SERS activity is originated from the electromagnetic field enhancements on the individual Mo nanoscrew, which is corroborated by single-particle optical characterizations
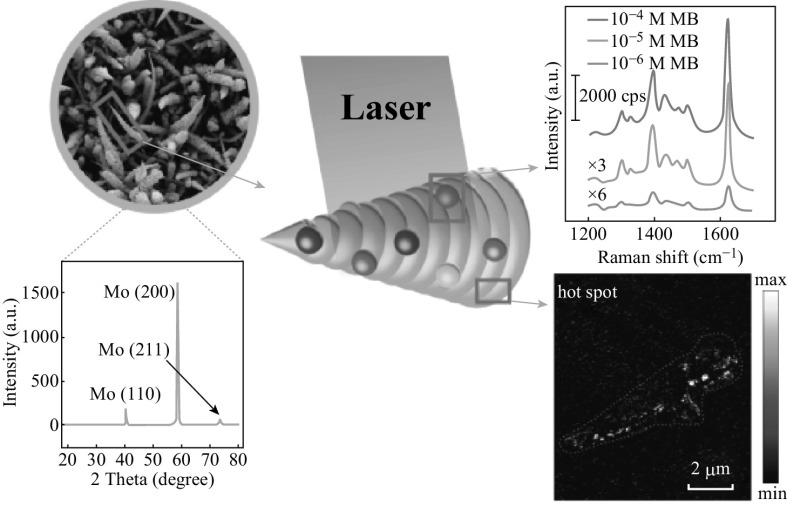

**Electronic supplementary material:**

The online version of this article (doi:10.1007/s40820-016-0104-6) contains supplementary material, which is available to authorized users.

## Introduction

Raman spectroscopy is well known as a noninvasive, non-labeling, and fingerprint-type sensing technique, which has been widely utilized in environmental and life science sensing and diagnostics [1–4]. However, traditional Raman spectroscopies usually suffer from low sensitivity due to the small Raman scattering cross sections of the analytes. The discovery and development of SERS has brought renascence to the Raman spectroscopy in terms of remarkable enhanced Raman intensity from the analytes [5–8]. The ultrasensitive sensing ability of the SERS is primarily associated with the enhanced electromagnetic field experienced by the analyte molecules in the vicinity of nanostructures with light focusing property [9–11]. Coinage metal nanostructures are the most studied architectures as SERS substrates [6, 12–15]. These metallic nanostructures can sustain plasmon resonances covering a broad spectral range, giving rise to strong near field electromagnetic field enhancement in their nearby regions under resonance excitation. Such field enhancement can strengthen the Raman scattering of the analyte molecules due to the fourth-power-dependence of the Raman intensity on the incoming electric field.

On the other hand, in recent years people begin to show great interests in non-coinage metal-based nanostructures with SERS activity. Such nanostructures can benefit the design and fabrication of high-performance sensors with multiple functionalities that are derived from different materials. For example, dielectric semiconductor nanostructures with high refractive index, such as Si, ZnO, TiO_2_, Fe_2_O_3_ [16–18], have been shown to exhibit intriguing optical field enhancement properties and have attracted many research interests as novel SERS substrates. In comparison with their coinage metal counterparts, the dielectric nanostructures usually exhibit excellent thermal stability and biocompatibility.

Another type of materials that are considered to exhibit SERS activity is transition metals [19–23]. Due to their unique *d*-band electrons, the transition metals can efficiently intermediate various chemical reactions under proper electric bias, which makes them excellent catalysis in various electrochemical reactions. It is therefore possible to combine the SERS and electrocatalytic behaviors of the transition metals as powerful manner to in situ monitor the interfacial reactions. In these regards, various transition metals, such as Ni, Fe, Ru, and Co, have been cultivated as SERS substrates since the 1990s [19–24]. Some of them have even been utilized for investigating the adsorption behavior of organic molecules [23], opening up new avenues for designing of novel surface characterization techniques.

To the best of our knowledge, most of the previous studies on transition metal-based SERS substrates focused on thin films with nanostructured roughness while leaving the elongated nanostructures less explored [25, 26]. In comparison with other geometries, the elongated nanostructures usually exhibit stronger electric field enhancements. However, up to now there is still a lack of consensus on the mechanisms governing the SERS activity of the transition metal nanostructures, which demand for systematic investigations. On one hand, previous studies using transition metal films with nanostructured surfaces suggested that due to the unique electronic band structures of the transition metals, charge transfer could happen between the analyte molecules and metal surface. As a result, the molecules can be strongly polarized and give rise to enhanced Raman scattering. This is the so-called chemical enhancement mechanism [19–24]. On the other hand, the transition metal nanostructures should in principle localize the electromagnetic field closely to their surfaces, which can lead to strong electromagnetic field enhancement at their vicinities. The Raman scattering of the adsorbed molecules will therefore be enhanced. This is the electromagnetic enhancement mechanism, which is well studied in noble metal nanostructures [12–15, 27].

Here, we demonstrate the observation of SERS activity from quasi-one-dimensional crystalline molybdenum (Mo) nanoscrew assemblies. The as-prepared Mo nanoscrews consist of many screw threads on their surfaces and very sharp heads at the ends. Such geometry is capable of concentrating the light field to enhance the Raman scattering of the adsorbed methylene blue (MB) molecules. Raman mapping indicated that the nanoscrew substrate exhibited reproducible SERS activity over an area of 100 × 100 μm^2^. The underlying mechanisms of the SERS behavior of the Mo nanoscrew assemblies were then systematically explored in terms of single-particle dark-field scattering spectroscopy, scanning near-field optical microscopy (SNOM), and numerical electromagnetic simulations, which unveiled that the amplified Raman signal were associated with the electric field enhancement originated from the optical coupling between the screw threads in each individual nanoscrew. Besides, the electromagnetic coupling between different nanoscrews can additionally contribute to the SERS activity of the nanoscrew assemblies. The results obtained in our study can not only demonstrate the SERS activity of the Mo nanostructures, but also further our understanding on light-matter interactions at the nanoscale.

## Experimental Sections

### Preparation of the Mo Nanoscrews

A Mo boat and a stainless steel substrate were separated with 3 mm spacing and placed in the center of a vacuum chamber. Gas mixture of argon and hydrogen was introduced into the chamber when the vacuum in the chamber was below 5 × 10^−2^ Torr. Thereafter, the Mo boat was heated up to 1350 °C at a rate of 50 °C min^−1^. For obtaining the Mo nanoscrews, the steel substrate was kept at 1350 °C for another 15 min followed by a natural cooling process [28].

### Characterizations

Scanning electron microscope (SEM) images were conducted using Zeiss Supra55 microscopes. Transmission electron microscope (TEM) image was obtained by JEOL JEM-2010 microscope operating at 200 kV. Electron dispersive spectrum (EDX) analysis was performed using INCAX-max 250 analyzer integrated within the Zeiss Supra55 microscope. Powder X-ray diffraction (XRD) analysis was conducted using a Rigaku RINT2400 X-ray diffractometer.

Dark-field scattering spectroscopy and imaging were conducted using a home-made dark-field spectroscopy system integrated with a white-light quartz tungsten halogen lamp as excitation source [29]. The scattered spectra from the Mo nanoscrews were corrected by first subtracting the background spectra taken from the adjacent regions without nanoscrews and then dividing them with the calibrated response curve of the entire optical system. Colored scattering images were captured using the color digital camera (ARTCAM-300MI-C, ACH Technology Co., Ltd., Shanghai) mounted on the imaging plane of the microscope.

### Sample Preparation for SERS Measurements

All of the four types of substrates, Mo nanoscrews, Mo thin film-coated glass, quartz, and stainless steel, were immersed in MB solution with specific molecule concentrations for about 4 h to allow for the adsorption of the molecules. The MB solution of various concentrations was prepared by dissolving different amounts of molecule powder into ethanol (99.9 vol%). Afterwards the substrates were dried in air. To prepare the sample for single-particle optical characterizations, the Mo nanoscrews were first scratched from the stainless steel where they were grown and then dispersed into the ethanol. The solution containing the nanoscrews was then subjected to ultrasonication for 10 min to disperse the nanoscrews thoroughly. Thereafter, 10 µL of the suspension was drop-casted onto the Mo- or ITO-coated glass with a pipet and then dried naturally under ambient conditions. The distribution of the nanoscrews onto the substrate can be controlled by tailoring the concentration of the nanoscrew dispersion, which was diluted until well-dispersed nanoscrews were found on the substrate. The substrates deposited with Mo nanoscrews were then immersed in MB solution (10^−4^ M) for about 4 h. The samples were dried in air at room temperature before the Raman measurements. A pattern-matching method was then utilized to locate the individual nanoscrew for both morphology and optical characterizations [30].

### Raman Measurements

The Raman spectra were acquired using a Renishaw inVia Reflex confocal micro-Raman system equipped with a Leica dark-field microscope. The excitation laser of 633 nm was focused onto the samples with a diameter of ∼1 μm through a 50× objective (Leica, numerical aperture: 0.8). The signal acquisition time for measuring the Raman scattering from samples adsorbed with MB molecules of 10^−4^, 10^−5^, and 10^−6^ M were 10, 30, and 60 s, respectively. Raman maps were obtained by scanning the samples under the microscope and recording the spectrum from every single point. For obtaining the maps with large area of 100 × 100 μm^2^, the scanning step was 1 μm. To obtain the maps of single Mo nanoscrews, the scanning step was 0.5 μm. The integrated intensity of the Raman band between 1550 and 1700 cm^−1^ was used to create the Raman maps.

### AFM and SNOM Characterizations

AFM and SNOM characterizations were conducted using a NeaSNOM scattering-type near-field optical microscope (Neaspec GmbH). In a specific measurement, an AFM tip coated with metal layer was illuminated using a visible laser with wavelength of 633 nm. The tip was vibrated vertically with frequency around 280 kHz. The backscattered light from the tip was detected using a pseudoheterodyne interferometric manner, where the scattered light was demodulated at third harmonic of the tip vibration frequency. The optical and topography images of the sample can be simultaneously obtained by scanning the sample below the tip.

### Numerical Calculations

The numerical calculations for the electromagnetic field distribution of an individual Mo nanoscrew were performed using commercial software package (COMSOL Multiphysics v4.3b). In a specific calculation, the screw threads were modeled as casks. The nanoscrew was modeled by stacking series of casks along the length direction of a free-standing tapered cylinder. For simplicity, the supporting substrate was not considered in the calculation. The length of the cylinder was 1.3 μm; the thickness of the casks was 100 nm, and the separation between adjacent casks was set as 2 nm. The dielectric function of Mo was employed according to previous measurements [31]. In the simulation, a *p*-polarized light with an incidence angle of 45° was launched into the box containing the Mo nanoscrew to simulate the interaction between the excitation light and the nanoscrew. The excitation wavelength was 633 nm.

## Results and Discussion

**Fig. 1:**
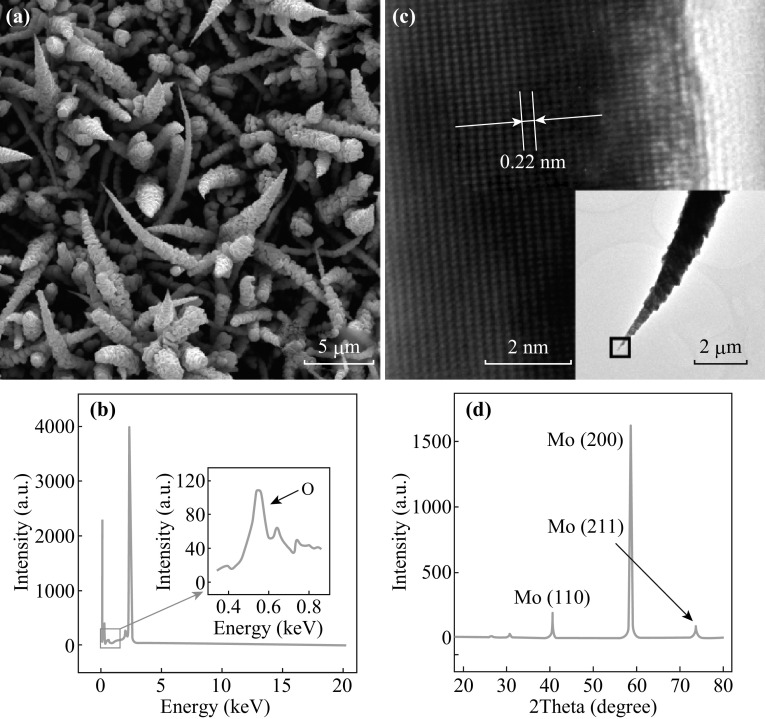
Morphology and composition characterizations of the Mo nanoscrews. **a** Representative SEM image of the Mo nanoscrews. **b** EDS spectrum of the Mo nanoscrews. *Inset* magnified EDS spectrum showing the oxygen peak. **c** TEM image of a typical Mo nanoscrew. *Inset* high-magnification TEM image of area enclosed by the square. **d** XRD spectrum of the Mo nanoscrews

Figure 1a shows a typical SEM image of the as-prepared sample, which indicates relatively uniform size and shape distributions. The average density of the nanoscrews is around 4–5 × 10^7^ cm^−2^. The lengths of the nanoscrew range from 2 to 6 μm, with maximum and minimum diameters of over 500 and 50 nm at their bottoms and tips, respectively. Each individual nanoscrew was consisted of many small screw threads stacking along the longitudinal direction with close separations. The EDS analysis of the nanoscrews indicated that they are mainly composed of metal Mo (Fig. 1b). One should note that small amount of oxygen, with an intensity ratio of ~1/40 to that of the Mo, shows up in the EDS spectrum (Fig. 1b, inset). Such a small amount of oxygen can be ascribed to contaminations during the measurements. In our experiment, before the EDS characterization the Mo nanoscrew was transferred from the growth chamber into the SEM chamber for the EDS measurement. During the transfer, the Mo nanoscrews were exposed to atmospheric conditions, whereby the oxygen and oxidized species (such as –OH) could be adsorbed onto the metal surface. Due to these contaminations, the oxygen peak will show up with weak intensity in the EDS spectrum. Besides, in the EDS measurements due to the limitation of the detector as well as collection geometry, usually light elements with atomic number less than 11 can be very difficult to measure reliably, if they can be detected at all [32]. Therefore, the oxygen peak observed from the EDS spectrum can only be used for reference while not for quantitative analysis.

The morphologies and crystal structure of the nanoscrews can be further revealed by the TEM imaging and XRD analysis (Fig. 1c, d). All of the diffraction peaks in the XRD profile can be indexed as body-centered cubic crystalline Mo (JCPDS 42-1120). The high-resolution TEM image indicated that the small screw threads on the nanoscrew were single-crystalline with a lattice spacing of 0.22 nm, which corresponded to the (110) plane of Mo. On the basis of these characterizations, we conclude that the nanoscrews are made up of pure metal Mo.

**Fig. 2:**
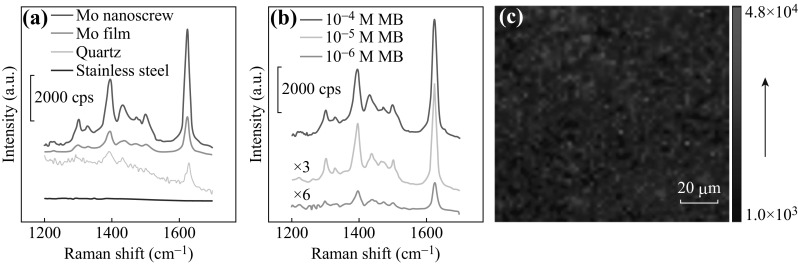
SERS activity characterizations of the Mo nanoscrews. **a** Raman spectra of MB molecules (10^−4^ M) adsorbed onto the Mo nanoscrews, Mo thin film, quartz, and stainless steel. The excitation wavelength was 633 nm. **b** SERS spectra of the Mo nanoscrews adsorbed with 10^−4^ M (*green*), 10^−5^ M (*orange*), and 10^−6^ M (*light blue*) of MB molecules. **c** Raman mapping image of the MB molecules (10^−4^ M) adsorbed onto the Mo nanoscrews. The mapping corresponded to the integrated intensity of the Raman band between 1550 and 1700 cm^−1^. The mapping area was over 100 × 100 μm^2^. (Color figure online)

The unique structures of the Mo nanoscrews were expected to be able to localize incidence light for enhancing the Raman scattering of the adjacent molecules. MB molecules were then utilized as probe analytes for assessing the SERS performance of the Mo nanoscrews. Besides the nanoscrew sample, three more types of substrates, including rough Mo thin film, quartz, and stainless steel, were used as references. The Mo thin film was prepared by magneto sputtering, which could lead to surface full of bumps and hollows. To evaluate the roughness, the AFM was employed to measure the topography of the Mo film. The root-mean-square average of height deviation on the as-prepared Mo thin film was ~3 nm (Fig. S1). Figure 2a gives the Raman spectra of the MB molecules adsorbed onto the four substrates under 633-nm laser excitation. Except the stainless steel, the other three substrates gave clear Raman bands from the MB molecules, which were assigned to *ν*_(CC)ring_ + *ν*_(CNC)ring_ mode (1622, 1397 cm^−1^) and *ν*_(CC)ring_ mode (1434 cm^−1^), respectively (Table 1) [33]. In comparison with the other two substrates, the Raman intensity was evidently enhanced for Mo nanoscrews adsorbed with MB molecules. These results suggested that owing to their polycrystalline surface features and porous architectures, the Mo nanoscrews demonstrated superior SERS activity than that of the rough Mo thin film.

**Table 1:** Band assignments of the Raman spectrum from the MB molecules [33]

Raman shift (cm^−1^)	Band assignment
1622	*ν*_(CC)ring_ + *ν*_(CNC)ring_
1500	*ν*_(CC)ring_
1468	*ν*_(CC)ring_
1434	*ν*_(CC)ring_
1397	*ν*_(CC)ring_ + *ν*_(CNC)ring_
1302	*ν*_(CC)ring_

We would like to discuss more on choose of the excitation wavelength for the Raman characterizations. In the Raman measurements, the Raman shifts of a specific type of molecule does not change with different excitation wavelengths. However, the intensities of the Raman bands are strongly dependent on the excitation wavelengths. On one hand, the intensity of the Raman scattering scales as 1/*λ*_ex_^4^, where the *λ*_ex_ is the excitation wavelength [34]. Hence excitation with a shorter wavelength can result in a stronger Raman scattering. On the other hand, when the excitation frequency is in resonance with an electronic transition of the molecule under investigation, the Raman scattering intensity of the molecule will be strongly enhanced [35]. In such a manner, choosing an excitation wavelength close to the absorption band of the molecule can strengthen the Raman intensity. In our study, the absorption band of the MB molecule centers around 650 nm (Fig. S2a). We therefore employed an excitation wavelength of 633 nm for the Raman characterizations for better demonstration. As a reference, we also conducted the Raman measurements using 785-nm laser as the excitation source, which is far from the MB absorption band. As expected, the Raman intensity of the MB is much lower than that under 633-nm excitation (Fig. S2b). However, in comparison with other substrates, the excellent SERS performance of the Mo nanoscrews still exists under the 785-nm excitation (Fig. S2c).

The SERS activity of the Mo nanoscrews was further studied by immersing the substrates into MB molecule solutions with various concentrations. Figure 2b shows that the Raman intensity degrades as the molecule concentrations are reduced. The main Raman bands of the MB molecules can still be distinguished when the MB concentration reaches 10^−6^ M. Such performance is much better than most reported values observed in transition metal-based SERS substrates [19–26]. One of the crucial parameters for characterizing the performance of a SERS substrate is the reproducibility of the Raman signal. To evaluate the SERS reproducibility of the Mo nanoscrews, we conducted the Raman mapping within a typical sample by monitoring the integrated intensity between 1550 and 1700 cm^−1^ over an area larger than 100 × 100 μm^2^ (Fig. 2c). The results clearly indicated that the Raman spectra collected from different regions were comparable with each other with a relative standard deviation value of ~0.3, suggesting the excellent SERS reproducibility of the Mo nanoscrews.

**Fig. 3:**
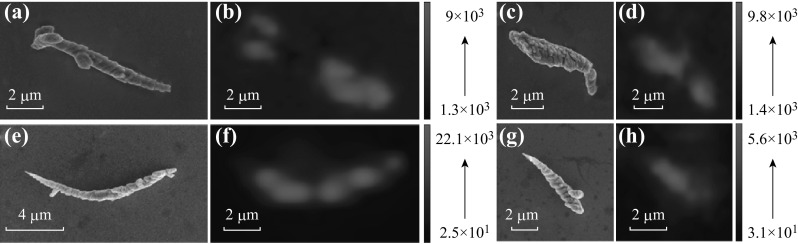
Raman mapping of the individual Mo nanoscrews modified with MB molecules (10^−4^ M). **a**–**d** SEM images and corresponding Raman maps of the Mo nanoscrews deposited onto the Mo film-coated glass substrate. **e**–**h** SEM images and corresponding Raman maps of the Mo nanoscrews deposited onto the ITO-coated glass substrate. The excitation wavelength was 633 nm. The mapping corresponded to the integrated intensity of the Raman band between 1550 and 1700 cm^−1^

To further reveal the origin of the SERS activity of the nanoscrews, Raman measurements were conducted on typical individual Mo nanoscrew. In a specific measurement, the Mo nanoscrews were transferred to the Mo-coated glass from the stainless steel substrate, which allowed for both optical measurements and SEM imaging. Another reason for choosing the Mo-coated glass as supported substrate was to provide direct comparison of the SERS performance between the Mo nanoscrews and Mo thin film. The SERS activity from the individual Mo nanoscrews can be apparently seen by comparing the SEM images and associated Raman maps (Fig. 3a–d). The distinct bright red regions with stronger Raman signal intensity showed similar shapes as the profiles of the Mo nanoscrews, suggesting that molecules adsorbed onto the nanoscrews exhibited stronger Raman intensities than those adsorbed onto the Mo thin film. These findings were consistent with the above ensemble measurements. In order to rule out the possibility that the Raman enhancement was originated from the strong coupling between the Mo nanoscrews and metallic Mo film with high refractive index, Raman mapping characterizations were also conducted for nanoscrews deposited onto indium tin oxide (ITO)-coated glass substrates (Fig. 3e–h) under the same excitation conditions. From the results, one can see that the SERS activity persisted for individual Mo nanoscrew deposited onto the ITO-coated glass substrate. These single-particle Raman mapping characterizations clearly indicated that the Raman enhancement behavior of the Mo nanoscrews was contributed from the intrinsic SERS activity of each nanoscrew in the assemblies.

For SERS substrate, the Raman enhancement factor (EF) is a pivotal parameter for quantitatively characterizing its SERS performance. However, in our study, it is difficult to precisely determine the EF of the nanoscrews substrate due to the random distribution of the nanoscrews in the assemblies and the irregular shape of each individual nanoscrew. To provide a preliminary estimation, we choose to calculate the EF using the results from the above single-particle measurements. Specifically, the EF of the substrate can be calculated by referring to the 1622 cm^−1^ mode of the MB molecule according the following equation [36, 37],

1 $${\text{EF}} = \frac{{I_{\text{SERS}} /N_{\text{SERS}} }}{{I_{\text{Raman}} /N_{\text{Raman}} }},$$

where *I*_Raman_ and *I*_SERS_ are the intensities of the 1622 cm^−1^ band for the normal (measured on the ITO-coated glass adsorbed with the MB molecules) and SERS (measured on an individual Mo nanoscrew adsorbed with the MB molecules) spectra, *N*_Raman_ is the number of molecules probed for a normal Raman scattering, and *N*_SERS_ is the number of molecules probed on the Mo nanoscrew. In our calculations, we employed the area intensity of the 1622 cm^−1^ band for both *I*_Raman_ and *I*_SERS_. We approximated that the molecules were uniformly adsorbed onto the ITO-coated glass and nanoscrew. Therefore, the ratio between the *N*_Raman_ and *N*_SERS_ can be replaced by the ratio between the areas of the ITO-coated glass (*A*_Raman_) and nanoscrew (*A*_SERS_) within the laser spot.

The *A*_Raman_ is the area of laser spot with a diameter of 1 µm. The *A*_SERS_ can be estimated according to the structure of the nanoscrew. We modeled the nanoscrew as stacking casks along its length direction (Fig. S3a, b). The surface area of each cask can be obtained using the following equation (Fig. S3c),

2 $$A_{\text{cask}} = \int {{\text{d}}A} = \int {2\pi x{\text{d}}s = } 2\int_{R}^{R + r} {\frac{{2\pi rx{\text{d}}x}}{{\sqrt {r^{2} - \left( {x - R} \right)^{2} } }} = 2\left( {\pi^{2} rR + 2\pi r^{2} } \right)}.$$

The *A*_SERS_ can then be expressed as

3 $$A_{\text{SERS}} = 0.5\,NA_{\text{cask}},$$

where *N* is the number of casks within the laser spot. The factor 0.5 is due to the fact that only half of the cask is exposed to the laser. According to the SEM characterizations (Fig. 1a), we chose *R* and *r* as 200 and 50 nm, respectively. There are about 10 casks were illuminated by the laser with a spot size of 1 μm in diameter. The ratio of the Raman intensity between the individual Mo nanoscrew and ITO-coated glass was ~1000 (Fig. 3f). Therefore, the EF of an individual Mo nanoscrew can be calculated to be 700.

**Fig. 4:**
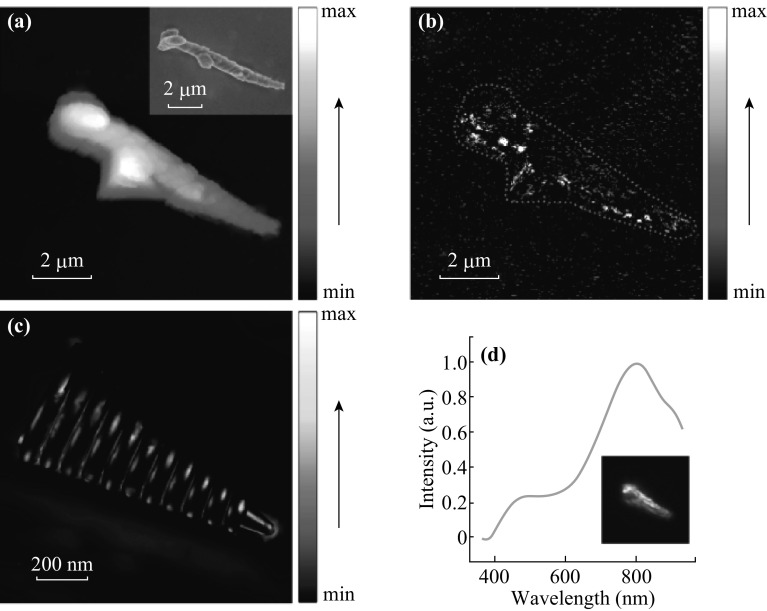
Characterizations of the hot-spots on an individual Mo nanoscrew. **a** AFM topography image of a typical Mo nanoscrew. *Inset* corresponding SEM image of the nanoscrew. **b** Optical near-field amplitude (3rd harmonics) at excitation of 633 nm recorded in the same area as that in **a**. **c** Calculated electric-field-magnitude enhancement contour of an individual Mo nanoscrew. The nanoscrew was excited by a *p*-polarized light at an incidence angle of 45°. The polarization of the incidence light had a component parallel to the length direction of the nanoscrew. The excitation wavelength was 633 nm. **d** Dark-field scattering spectrum of the Mo nanoscrew. *Inset* dark-field scattering image of the Mo nanoscrew

As mentioned above, the hot-spots with strong electromagnetic field enhancements are very important for the SERS activity of a specific substrate. To unveil the hot-spots in the Mo nanoscrews, we employed single-particle SNOM imaging technique to measure the electromagnetic near field distributions within an individual nanoscrew. The measurements were based on apertureless scattering-type SNOM (*s*-SNOM), whereby simultaneous morphology and near-field optical images can be obtained via scanning the sample under the atomic force microscope (AFM) tip [38, 39]. Typical topography and associated near-field optical images of a Mo nanoscrew were shown in Fig. 4a, b. Screw threads stacking along the longitudinal direction of the nanoscrew can be seen from the topography image, while the optical image was manifested by bright spots of enhanced optical amplitudes with respect to the background. These regions were corresponded to the hot-spots and not ubiquitous on the nanoscrew. Due to fact that both of the SEM (Fig. 4a, inset) and AFM images had indicated small separations between the screw threads on the nanoscrew, we attributed these hot-spots to the near-field optical coupling between adjacent screw threads, which was similar to those formed between noble metal nanoparticles with small separations [27]. To prove this, numerical electromagnetic simulation was performed to elucidate the electric field distributions on the Mo nanoscrew. To simplify the analysis without losing the physics, in the calculations, the screw threads were modeled as casks. The nanoscrew was modeled by stacking series of casks along the length direction of a free-standing tapered cylinder (Fig. 4c). The relatively large radius of the AFM tip precluded determination of the separation between adjacent screw threads. Therefore, we arbitrarily chose a small value of 2 nm for the separations between adjacent casks in a specific calculation. The calculated near field distributions evidently showed that the gap regions between two neighboring casks could confine the electric field to form hot-spots (Fig. 4c). Furthermore, the sharp tip of the nanoscrew was also shown to strongly concentrate the incidence light. These results agreed with the previous experimental measurements.

The strong hot-spots can be further manifested by the far-field scattering properties of the nanoscrews. Due to the localized enhanced electric field, the nanoscrew can be strongly polarized. The oscillating polarized charges in the nanoscrew can therefore induce strong light scattering into the far-field, giving rise to enhanced scattering bands in the visible and near-infrared regions (Fig. 4d). Such strong light scattering behavior of the nanoscrew can be also seen from its scattering image, which shows vivid color appearance (Fig. 4d, inset). These responses of the nanoscrew also suggest its potential in future visible and near-infrared photonic and optoelectronic applications.

The optical near-field results clearly reveal the hot-spots in an individual Mo nanoscrew, which are responsible for the SERS activity of the nanoscrew assemblies. On the other hand, in the assembly the separations between different nanoscrews are small (Fig. 1a), whereby effective electromagnetic coupling between adjacent Mo nanoscrews can be induced. Such coupling effects can give rise to even stronger electric field enhancements, which will additionally contribute to the Raman enhancements of the nanoscrew assemblies. In our current study, we intend to demonstrate the Mo nanoscrews as a novel type of SERS substrate and elucidate the underlying mechanisms. In this regard, it is more important for us to characterize the intrinsic SERS activity from an individual Mo nanoscrew and evaluate its ability to localize the electromagnetic field. Therefore, we did not pay much attention to optimize the SERS performance by taking advantage of the coupling between different nanoscrews in the assemblies.

## Conclusion

In conclusion, we have cultivated the Mo nanoscrews as non-coinage-metal SERS substrate. By using Raman mapping technique, we have elucidated the SERS activity of the nanoscrews with good reproducibility. The SERS effect is attributed to the intrinsic SERS activity of each nanoscrew in the assembly. Through high-resolution SNOM and SEM measurements, we have shown that the Raman enhancement behavior of the individual nanoscrew is associated with its unique structure. The as-prepared Mo nanoscrews were composed of many small screw threads stacking along the longitudinal direction with small separations. Such small gaps can act as hot-spots to localize the incidence light for strong electric field enhancements, giving rise to the observed SERS activity. We strongly believe that the results obtained in this study can on one hand help extend the SERS substrates to other low-cost and stable material systems, and on the other hand fertilize the functionalities of transition metal-based nanostructures.

## Electronic supplementary material

Below is the link to the electronic supplementary material.

Supplementary material 1 (PDF 290 kb)
