# Supplementary material for: Molybdenum Nanoscrews: A Novel Non-coinage-Metal Substrate for Surface-Enhanced Raman Scattering
Source: Nanomicro Lett. 2016 Aug 31;9(1):2. doi: 10.1007/s40820-016-0104-6 (PMC6223773; doi:10.1007/s40820-016-0104-6)
Supplement: Supplementary file 1 — Supplementary material 1 (PDF 290 kb) [file 40820_2016_104_MOESM1_ESM.pdf]

Supplementary Information for

Molybdenum Nanoscrews: A Novel Non-Coinage-Metal Substrate for Surface-Enhanced Raman Scattering

Di An, Yan Shen, Jinxiu Wen, Zebo Zheng, Jun Chen, Juncong She, Huanjun Chen*, Shaozhi Deng, Ningsheng Xu*

State Key Laboratory of Optoelectronic Materials and Technologies, Guangdong Province Key Laboratory of Display Material and Technology, School of Electronics and Information Technology, Sun Yat-sen University, Guangzhou 510275, People's Republic of China

*****Corresponding authors. chenhj8@mail.sysu.edu.cn, stsxns@mail.sysu.edu.cn

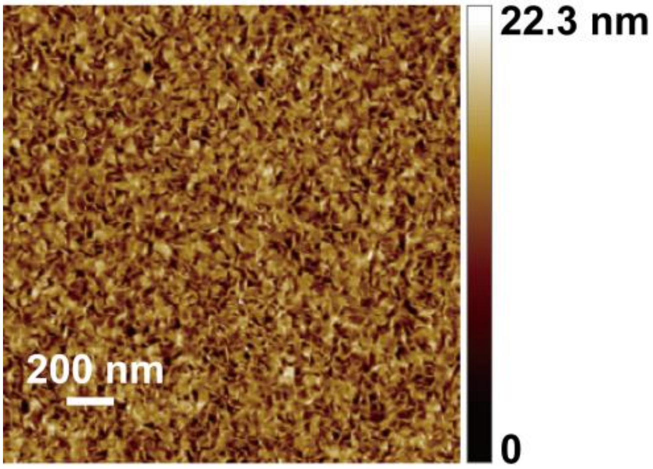

Fig. S1 AFM topography of the as-prepared Mo thin film.

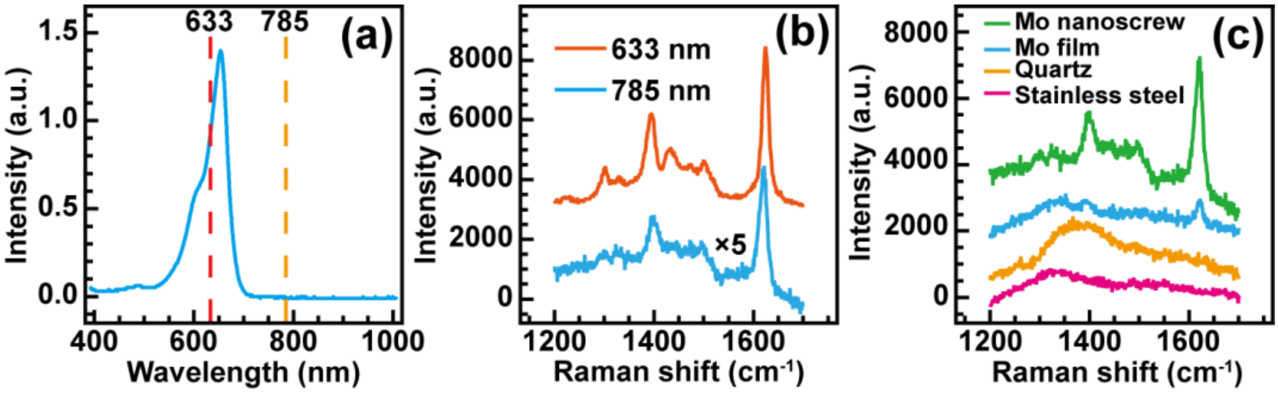

Fig. S2 Raman characterizations using the 785-nm laser as the excitation source. **a** Optical absorption spectrum of the MB molecule. The excitation laser wavelengths at 633 nm and 785 nm are indicated by the dashed lines, respectively. **b** Comparison of the Raman spectra of MB molecules (10^{-4} M) adsorbed onto the Mo nanoscrews using excitation wavelengths of 633 nm and 785 nm. **c** Raman spectra of MB molecules (10^{-4} M) adsorbed onto the Mo nanoscrews, Mo thin film, quartz, and stainless steel. The excitation wavelength was 785 nm

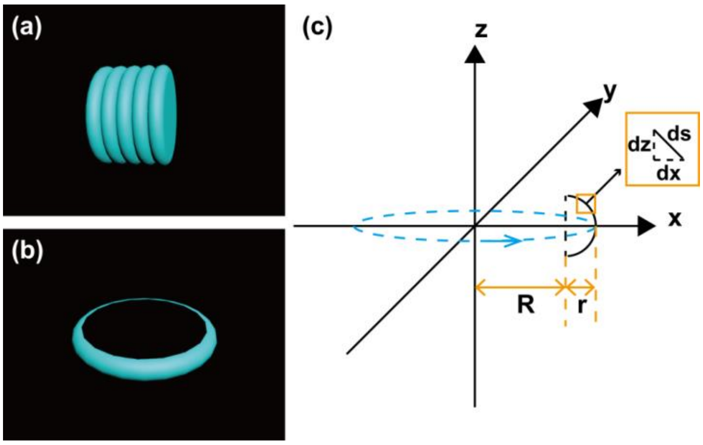

Fig. S3 **a** Schematic showing the structure of an individual Mo nanoscrew, where the nanoscrew is modeled as casks stacking along its length direction. **b** Close view of a cask. **c** Scheme for the calculation of the surface area of an individual cask
